# Fatigue Across the Lifespan in Men and Women: State vs. Trait

**DOI:** 10.3389/fnhum.2022.790006

**Published:** 2022-05-09

**Authors:** Glenn R. Wylie, Amanda J. Pra Sisto, Helen M. Genova, John DeLuca

**Affiliations:** ^1^Kessler Foundation, Rocco Ortenzio Neuroimaging Center, West Orange, NJ, United States; ^2^Department of Physical Medicine and Rehabilitation, New Jersey Medical School, Rutgers University, Newark, NJ, United States; ^3^Department of Veterans’ Affairs, War Related Illness and Injury Study Center, New Jersey Healthcare System, East Orange, NJ, United States; ^4^Department of Neurology, New Jersey Medical School, Rutgers University, Newark, NJ, United States

**Keywords:** cognitive fatigue, aging, gender, fMRI, state fatigue, trait fatigue

## Abstract

**Objective:**

Fatigue is commonly thought to worsen with age, but the literature is mixed: some studies show that older individuals experience more fatigue, others report the reverse. Some inconsistencies in the literature may be related to gender differences in fatigue while others may be due to differences in the instruments used to study fatigue, since the correlation between state (in the moment) and trait (over an extended period of time) measures of fatigue has been shown to be weak. The purpose of the current study was to examine both state and trait fatigue across age and gender using neuroimaging and self-report data.

**Methods:**

We investigated the effects of age and gender in 43 healthy individuals on self-reported fatigue using the Modified Fatigue Impact Scale (MFIS), a measure of trait fatigue. We also conducted fMRI scans on these individuals and collected self-reported measures of state fatigue using the visual analog scale of fatigue (VAS-F) during a fatiguing task.

**Results:**

There was no correlation between age and total MFIS score (trait fatigue) (*r* = –0.029, *p* = 0.873), nor was there an effect of gender [*F*_(1,31)_ < 1]. However, for state fatigue, increasing age was associated with less fatigue [*F*_(1,35)_ = 9.19, *p* < 0.01, coefficient = –0.4]. In the neuroimaging data, age interacted with VAS-F in the middle frontal gyrus. In younger individuals (20–32), more activation was associated with less fatigue, for individuals aged 33–48 there was no relationship, and for older individuals (55+) more activation was associated with more fatigue. Gender also interacted with VAS-F in several areas including the orbital, middle, and inferior frontal gyri. For women, more activation was associated with less fatigue while for men, more activation was associated with more fatigue.

**Conclusion:**

Older individuals reported less fatigue during task performance (state measures). The neuroimaging data indicate that the role of middle frontal areas change across age: younger individuals may use these areas to combat fatigue, but this is not the case with older individuals. Moreover, these results may suggest greater resilience in females than males when faced with a fatiguing task.

## Introduction

While some qualities improve as we grow older (e.g., wisdom), other physical and mental abilities tend to decline later in life. For example, physical strength and processing speed both begin to wane as we pass middle age. Because sleep quality is another factor that commonly deteriorates with age (Åkerstedt et al., 2018), it might seem intuitive to expect fatigue to increase as age advances past middle age, but surprisingly the literature is mixed. Some studies have reported a greater prevalence of fatigue in the aged than in the young (Soyeur and Senol, 2011), while others have shown a decrease in fatigue with greater age (Åkerstedt et al., 2018).

While there is no clear, universally accepted definition of fatigue, it is widely agreed that fatigue is a state that is caused by exertion that results in changes in strategy or resource use (Kluger et al., 2013; Phillips, 2015). Fatigue also has several different subtypes, including cognitive or mental fatigue, psychological fatigue, and physical fatigue. The study described here is aimed at better understanding cognitive fatigue. Additionally, fatigue can be thought of as a trait—conceptualized as the extent to which one is prone to fatigue—or a state—the instantaneous experience of fatigue. While terms such as “fatigability” have been proposed to describe one’s propensity to become fatigued (Zijdewind et al., 2016), we prefer the state vs. trait distinction, given the long history of state and trait measures in the neuropsychological literature [e.g., Spielberger et al. (1970), Endler and Parker (1990), and Chiappelli et al. (2014)].

Fatigue is a troubling symptom in neurological disorders such as Multiple Sclerosis, but it is also widespread in the general population, with a prevalence of up to 45% in healthy individuals (Junghaenel et al., 2011; Galland-Decker et al., 2019). Much of the existing literature on fatigue points to fatigue differentially affecting younger vs. older populations, as well as males vs. females. Sleep problems and fatigue are often linked, such that those who get less sleep experience greater fatigue (Avlund, 2010). Several studies point to quality of sleep decreasing with age (Sivertsen et al., 2009; Mander et al., 2017), which suggests that fatigue may increase with age. However, fatigue and sleep are distinct constructs, and fatigue has not been found to consistently increase with age in the literature. One potential explanation for mixed findings in the literature examining age and fatigue is that different studies have used different fatigue assessments. For instance, Åkerstedt et al. (2018) used a “state” measure of fatigue (assessing subjects’ instantaneous experience of fatigue at the time of testing) and found that fatigue declined with age, even despite age-related increases in sleep problems. Conversely, Soyeur and Senol (2011) used a “trait” measure of fatigue (assessing subjects’ assessment of how much fatigue they had experienced over the previous four weeks) and discovered a high prevalence of fatigue in elderly people living in a rest home. Furthermore, in a study looking at fatigue in both cancer survivors and general populations using a trait fatigue measure, older participants (>64) reported greater fatigue than younger participants (Butt et al., 2010).

Another potential source of variance in the literature is gender. A few studies have found women to report greater trait levels of fatigue than men (Butt et al., 2010; Engberg et al., 2017). Biological factors, including menstruation and pregnancy, compounded with social context (e.g., taking care of young children), may result in elevated fatigue in females as opposed to males (Bensing et al., 1999). Avlund (2010) used trait measures to show that women reported more fatigue than men, but only when they were 70 years old or older. This suggests that age may interact with gender such that men and women show different propensities to trait fatigue across the lifespan. However, whether this also is found with state fatigue measures is currently unknown.

Here, we investigated the effects of age on both state and trait measures of fatigue, while also looking at the effects of gender. To study state fatigue, we induced cognitive fatigue by asking participants to repeatedly perform a demanding working memory paradigm (the 2-back condition of the N-back paradigm). We have used this approach to induce fatigue in healthy and clinical populations in previous work (Wylie et al., 2017a,^2019^; Chen et al., 2020b). We also included neuroimaging metrics—acquired during the fatigue induction task—because we have found these metrics to be important indexes of fatigue in other work (Wylie et al., 2017b,^2020^, ^2021^). Based on pre-existing literature, we hypothesized that trait measures of fatigue would show increased fatigue with age, while state measures would result in decreasing fatigue with age. We also hypothesized that women would report more fatigue than men on both state and trait fatigue measures. While there is evidence to suggest that older adults and younger adults may rely on different brain regions when performing a cognitive task (Crowell et al., 2020), there are no neuroimaging studies to our knowledge that have looked at the relationship between cognitive fatigue and brain activity across different ages. The ventromedial prefrontal cortex, the dorsal anterior cingulate cortex, the dorsolateral prefrontal cortex, and the anterior insula are regions that have been linked to cognitive fatigue [e.g., Wylie et al. (2019, 2020) and Chen et al. (2020a,b)], so we expected differences in activation in these areas between younger and older individuals, as well as between males and females. In order to better understand the neural substrates of age and gender on fatigue, we investigated where in the brain increasing levels of fatigue correlated with brain activation, accounting for age and gender.

## Materials and Methods

### Subjects

This study included 43 subjects. They ranged in age from 20 to 63, and 19 (44%) of them were women. The subjects were matched on age, education and gender distribution (see Table 1). Ten participants were aged 20–35, 21 participants were aged 36-50, and twelve participants were aged 51–63 (see Supplementary Figure 1 for the distribution of age across the sample). In order to be included in the study, participants were: aged 18–65; right handed; had normal or corrected-to-normal visual acuity; were native English speakers. Exclusion criteria were: a history head injury, stroke, seizures, or any other significant neurological history; a history of drug or alcohol abuse; clinically significant psychiatric history (such as schizophrenia or bipolar disorder); MRI contraindications; psychoactive medication use. All subjects provided informed consent to participate, in accordance with the Institutional Review Boards of Rutgers University and Kessler Foundation, and all were paid $100 for their participation.

**TABLE 1:** The demographics of the sample.

	Female (*N* = 19)	Male (*N* = 24)	Overall (*N* = 43)
**Age**			
Mean (SD)	40.1 (12.7)	44.6 (10.5)	42.6 (11.6)
Median [Min, Max]	37.0 [24.0, 59.0]	43.5 [20.0, 63.0]	42.0 [20.0, 63.0]
**Education**			
Mean (SD)	15.9 (2.17)	15.3 (2.36)	15.6 (2.28)
Median [Min, Max]	16.0 [12.0, 20.0]	16.0 [11.0, 21.0]	16.0 [11.0, 21.0]
**MFIS.total**			
Mean (SD)	11.4 (9.10)	10.9 (13.6)	11.2 (11.5)
Median [Min, Max]	11.0 [0, 28.0]	5.00 [0, 40.0]	8.00 [0, 40.0]

*Mean values are shown along with the associated standard deviation (SD); median values are also shown along with the corresponding minimum and maximum scores.*

#### Trait Fatigue Measure

We used the MFIS (Modified Fatigue Impact Scale) to assess trait fatigue, and in all cases the MFIS was acquired prior to the induction of state fatigue. The MFIS is a self-report instrument consisting of 21 items that evaluate how fatigue has impacted the lives of participants during the past 4 weeks. The MFIS total score ranges from 0 to 84 and is broken down into three subscales: physical (0–36), cognitive (0–40), and psychosocial (0–8) (Larson, 2013). Answers fall on a 5-point Likert scale ranging from “never” to “always.”

#### Visual Analog Scale of Fatigue

To evaluate the level of state fatigue, participants were presented with a visual analogue scale (VAS) before and after each block of the 2-back task. Participants were asked: “How mentally fatigued are you right now?” and were asked to indicate their level of fatigue using the response box on a scale from 0 to 100, with 0 being not at all fatigued and 100 being extremely fatigued. In order to mask the purpose of the study, six VASs were administered, in randomized order, before and after each task block: fatigue, happiness, sadness, pain, tension and anger.

#### Neuroimaging Acquisition

Neuroimaging data collection began on a 3-Tesla Siemens Allegra scanner (28 subjects) and was completed on a 3-Tesla Siemens Skyra scanner (15 subjects). For this reason, a regressor for scanner was included in all group-level analyses, as has been done in previous research utilizing more than one scanner (Stonnington et al., 2008; Biswal et al., 2010; Wylie et al., 2018). A T2*-weighted Echo Planar sequence was used to collect functional images during four blocks of task-related data collection, with 140 brain volume acquisitions per block (Allegra: echo time = 30 ms; repetition time = 2,000 ms; field of view = 22 cm; flip angle = 80°; slice thickness = 4 mm, 32 slices, matrix = 64 × 64, in-plane resolution = 3.438 × 3.438 mm^2^; Skyra: echo time = 30 ms; repetition time = 2000 ms; field of view = 22 cm; flip angle = 90°; slice thickness = 4 mm, 32 slices, matrix = 92 × 92, in-plane resolution = 2.391 × 2.391 mm^2^). A high-resolution magnetization prepared rapid gradient echo (MPRAGE) image was also acquired (Allegra: TE = 4.38 ms; TR = 2000 ms, FOV = 220 mm; flip angle = 8°; slice thickness = 1 mm, NEX = 1, matrix = 256 × 256, in-plane resolution = 0.859 × 0.859 mm^2^; Skyra: TE = 3.43 ms; TR = 2100 ms, FOV = 256 mm; flip angle = 9°; slice thickness = 1 mm, NEX = 1, matrix = 256 × 256, in-plane resolution = 1 × 1 mm^2^), and was used to register the functional data into standard MNI space for group analysis.

#### Behavioral Paradigm and Data

E-Prime software (Schneider et al., 2002) was used for behavioral data acquisition and stimulus presentation. During the four fMRI scans, participants were presented with the 2-back condition of the N-back working memory task—a demanding working memory task (Meule, 2017). Prior to scanning, all subjects practiced the task to criterion (80% correct) to ensure a comparable level of proficiency. In all cases, subjects reached criterion in a single block of 65 trials of the task. The sequence used for practice was not repeated during the fatigue induction session in the scanner. In each of the four blocks of the 2-back task used to induce fatigue inside the scanner there were 65 trials. On each trial a single letter was presented on the screen and participants were asked to respond by pressing a button on a response box every time the letter was the same as the letter presented two trials before (e.g., R N Q N…). Letters were presented in white (Arial 72 point font) on a black background. Of the 26 letters in the English alphabet, the following were used with equal frequency: A B C D F H J K M N P Q R S T V Z. The other letters were excluded to enhance the discriminability of the letters used. The letter stimuli remained on the screen for 1.5 s, followed by a 500 ms inter-trial interval (ITI), and the time between successive trials was jittered to allow for the data to be deconvolved as an event related design. The jittering was optimized using the Optseq2 program,^1^ and was achieved by inserting between zero and six null events between successive trials. The duration of each null event was a multiple of the length of the trial (2 s), drawn from a distribution following a power function. The majority of inter-trial intervals were 500 ms (zero null events), followed by 2 s (one null event) and so on. The average ITI was 1587.87 ms (±1769.7).

In order to ensure comparable stimulation across subjects, the stimuli remained on the screen for the full 1.5 s on each trial and were not removed when subjects responded. Each run lasted the same amount of time (280 s). The average amount of time between successive blocks was 2 min 04 s (S.D. = 2 min 17 s).

The outcome variables from the behavioral task were response time (RT) and accuracy. Trials were considered correct when the subject withheld a response on trials when a non-target letter was presented or when s/he responded with a latency longer than 150 ms on trials when a target was presented. For the analysis of the fMRI data, only trials on which a correct response (or correctly withheld response) were included.

### Analyses

#### Modified Fatigue Impact Scale

For the MFIS data, a bivariate correlation was run in SPSS to determine the association between age and total MFIS score, and between age and each MFIS subtype using Pearson’s r. One-way ANOVAs were run to analyze whether MFIS total, MFIS physical, MFIS cognitive, and MFIS psychological scores differed by gender (male vs. female).

#### Visual Analog Scale of Fatigue

For the analysis of the visual analog scale of fatigue (VAS-F) scores, a Linear Mixed Effects analysis (LME; using the R statistical package [version 3.4.3]) was used. Gender (male vs. female) was a between-subjects factor, and Rating (ratings 1–5) was a fixed effect. Age was a quantitative variable, and subject was included as a random factor.

Because VAS-F scores were obtained before and after each task block, the amount of fatigue during each block was estimated by using the mean of the scores before and after the relevant block; this value was used in the correlational analyses. Furthermore, because the VAS-F scores were skewed, they were transformed using the Box-Cox method to ensure that assumptions of normality were not violated (Box and Cox, 1964). The Box-Cox method is a power transformation in which a range of power transformations are considered and the one that best transforms the data into a normal distribution is selected.

#### Response Time and Accuracy

Response time and accuracy were analyzed with an LME that included the factors of Gender (female vs. male) and Run (runs 1–4); the VAS-F scores and age were included as quantitative variables; subject was included as a random factor.

#### Neuroimaging

Results included in this manuscript come from preprocessing performed using *fMRIPrep* 1.4.1 [Esteban et al. (2019); RRID:SCR_016216], which is based on *Nipype* 1.2.0 [Gorgolewski et al. (2011); RRID:SCR_002502].

For anatomical preprocessing, the T1-weighted (T1w) image from each subject was corrected for intensity non-uniformity (INU) with N4BiasFieldCorrection (Tustison et al., 2010), distributed with ANTs 2.2.0 [Avants et al. (2008); RRID:SCR_004757], and used as T1w-reference throughout the workflow. The T1w-reference was then skull-stripped with a *Nipype* implementation of the antsBrainExtraction.sh workflow (from ANTs), using OASIS30ANTs as target template. Brain tissue segmentation of cerebrospinal fluid (CSF), white-matter (WM), and gray-matter (GM) was performed on the brain-extracted T1w using fast [FSL 5.0.9, RRID:SCR_002823, Zhang et al. (2001)]. Volume-based spatial normalization to one standard space (MNI152NLin2009cAsym) was performed through non-linear registration with antsRegistration (ANTs 2.2.0), using brain-extracted versions of both T1w reference and the T1w template. The following template was selected for spatial normalization: *ICBM 152 Non-linear Asymmetrical template version 2009c* [Fonov et al. (2009), RRID:SCR_008796; TemplateFlow ID: MNI152NLin2009cAsym].

For functional data preprocessing each of the 8 BOLD runs found per subject (across all tasks and sessions), the following preprocessing was performed. First, a reference volume and its skull-stripped version were generated using a custom methodology of *fMRIPrep*. The BOLD reference was then co-registered to the T1w reference using flirt [FSL 5.0.9, Jenkinson and Smith (2001)] with the boundary-based registration (Greve and Fischl, 2009) cost-function. Co-registration was configured with nine degrees of freedom to account for distortions remaining in the BOLD reference. Head-motion parameters with respect to the BOLD reference (transformation matrices, and six corresponding rotation and translation parameters) are estimated before any spatiotemporal filtering using mcflirt [FSL 5.0.9, Jenkinson et al. (2002)]. BOLD runs were slice-time corrected using 3dTshift from AFNI 20160207 [Cox and Hyde (1997), RRID:SCR_005927]. The BOLD time-series (including slice-timing correction when applied) were resampled onto their original, native space by applying a single, composite transform to correct for head-motion and susceptibility distortions. These resampled BOLD time-series will be referred to as *preprocessed BOLD in original space*, or just *preprocessed BOLD*. The BOLD time-series were resampled into standard space, generating a *preprocessed BOLD run in [“MNI152NLin2009cAsym”] space*. First, a reference volume and its skull-stripped version were generated using a custom methodology of *fMRIPrep*. Several confounding time-series were calculated based on the *preprocessed BOLD*: framewise displacement (FD), DVARS and three region-wise global signals. FD and DVARS are calculated for each functional run, both using their implementations in *Nipype* [following the definitions by Power et al. (2014)]. The three global signals are extracted within the CSF, the WM, and the whole-brain masks. Additionally, a set of physiological regressors were extracted to allow for component-based noise correction [*CompCor*, Behzadi et al. (2007)]. Principal components are estimated after high-pass filtering the *preprocessed BOLD* time-series (using a discrete cosine filter with 128s cut-off) for the two *CompCor* variants: temporal (tCompCor) and anatomical (aCompCor). tCompCor components are then calculated from the top 5% variable voxels within a mask covering the subcortical regions. This subcortical mask is obtained by heavily eroding the brain mask, which ensures it does not include cortical GM regions. For aCompCor, components are calculated within the intersection of the aforementioned mask and the union of CSF and WM masks calculated in T1w space, after their projection to the native space of each functional run (using the inverse BOLD-to-T1w transformation). Components are also calculated separately within the WM and CSF masks. For each CompCor decomposition, the *k* components with the largest singular values are retained, such that the retained components’ time series are sufficient to explain 50 percent of variance across the nuisance mask (CSF, WM, combined, or temporal). The remaining components are dropped from consideration. The head-motion estimates calculated in the correction step were also placed within the corresponding confounds file. The confound time series derived from head motion estimates and global signals were expanded with the inclusion of temporal derivatives and quadratic terms for each (Satterthwaite et al., 2013). Frames that exceeded a threshold of 0.5 mm FD or 1.5 standardized DVARS were annotated as motion outliers. All resamplings can be performed with *a single interpolation step* by composing all the pertinent transformations (i.e., head-motion transform matrices, susceptibility distortion correction when available, and co-registrations to anatomical and output spaces). Gridded (volumetric) resamplings were performed using antsApplyTransforms (ANTs), configured with Lanczos interpolation to minimize the smoothing effects of other kernels (Lanczos, 1964). Non-gridded (surface) resamplings were performed using mri_vol2surf (FreeSurfer).

The resulting data were then deconvolved. In the deconvolution, signal drift was modeled with a set of basis functions; the motion parameters were used as regressors of no interest. The regressors of interest were the correct trials of each block. Each block was deconvolved separately, and the coefficient of fit of the correct trials was entered into the group-level analysis.

In all cases, an LME was used (3dLME from the AFNI suite of processing tools) with Gender (male vs. female) and Run (runs 1–4) as a factors; the VAS-F scores and age were included as quantitative variables; subject was included as a random factor.

The results of these analyses were corrected for multiple comparisons by using an individual voxel probability threshold of *p* < 0.005 and a cluster threshold of 28 voxels (voxel dimension = 2.4 mm × 2.4 mm × 4 mm). Monte Carlo simulations, using 3dClustSim (version AFNI_17.2.16, compile date: September 19, 2017) showed this combination to result in a corrected alpha level of *p* < 0.05.

## Results

### Trait Fatigue: Modified Fatigue Impact Scale

There was no correlation found between age and total MFIS score (*r* = –0.029, *p* = 0.873), nor was there a correlation found between age and any of the MFIS subscales (physical *r* = 0.072, *p* = 0.689, cognitive *r* = –0.075, *p* = 0.678, psychological *r* = –0.176, *p* = 0.327). The difference in total MFIS scores between males (x̄= 10.94) and females (x̄= 11.38) was not significant [*F*_(1,31)_ < 1] (see Table 1). Males and females did not differ in the physical [*F*_(1,32)_ < 1], cognitive [*F*_(1,32)_ < 1], or psychological [*F*_(1,32)_ < 1] fatigue subfields.

### State Fatigue: Visual Analog Scale of Fatigue

**FIGURE 1:**
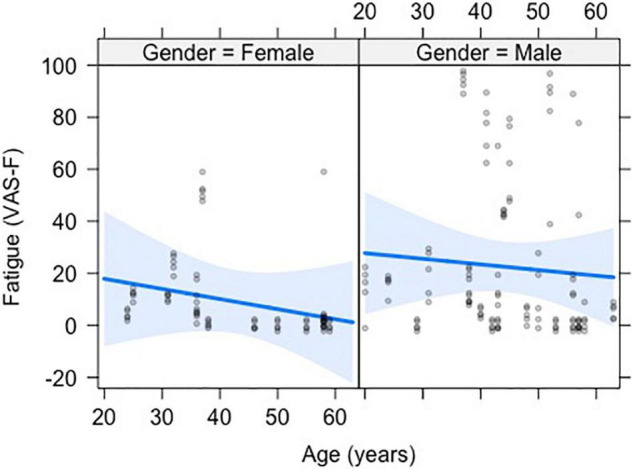
The negative relationship between visual analog scale of fatigue (VAS-F) and age is shown. The data from men and women are shown separately to demonstrate the similarity of the effect of age in the two groups. The blue line represents the best fitting linear relationship between VAS-F and age. The 95% confidence interval around this line is plotted in lighter blue. The data are represented by gray circles.

The analysis of state fatigue (VAS-F scores) showed a significant main effect of Age [*F*_(1,35)_ = 9.19, *p* < 0.01, η^2^_partial_ = 0.21], with a correlation of *r* = –0.20 and a negative coefficient of –0.401, meaning that for each year of increased age subjects reported 0.401 less fatigue on the VAS-F scale (see Figure 1). No other effects or interactions were significant. However, because prior research using this task has shown an effect of Rating (Wylie et al., 2019, 2020; Chen et al., 2020b), we computed the pairwise differences between ratings 2–5 relative to rating 1. The VAS-F scores reported for rating 5 (19.6) differed from rating 1 (18.4) [*t*_(152)_ = 2.07, *p* < 0.05, η^2^_partial_ = 0.03].

For all subsequent analyses, we analyzed only data from blocks/runs on which subjects reported at least some fatigue—that is, the fatigue reported was greater than zero. This resulted in the exclusion of runs from seven men (age range 29–57) and six women (age range 38–59). Subjects reported experiencing fatigue on 72% of the experimental runs, or on 145 out of 185 runs.

### Response Time and Accuracy

**FIGURE 2:**
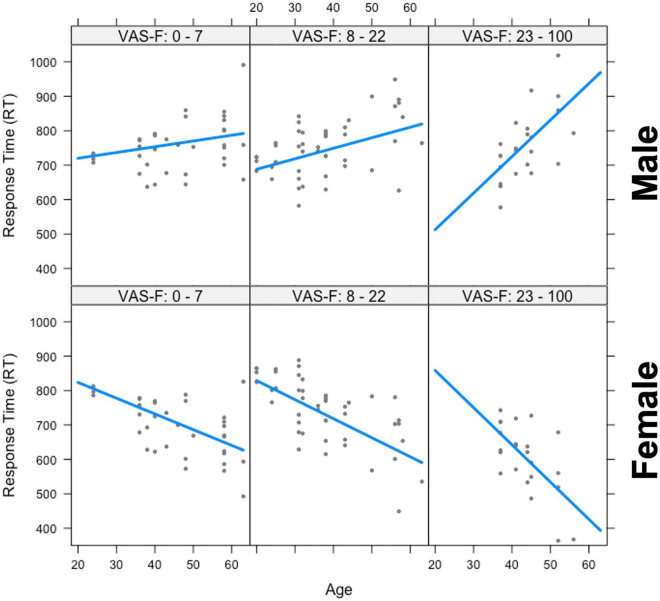
The interaction between age, gender, and visual analog scale of fatigue (VAS-F) on Response Time (RT). Age is plotted in years and RT is plotted in milliseconds. The top three panels show the positive relationship between RT and age for men and the bottom three panels show the negative relationship between RT and age for women across increasing values of VAS-F, indicated at the top of each panel. For the plot, the VAS-F data was divided into three bins for expository purposes only. In the analysis, the VAS-F data was continuous. The lines represents the best fitting linear relationship between RT and age.

The analysis of the response time (RT) data showed a significant main effect of Gender [*F*_(1,25.7)_ = 9.47, *p* < 0.01, η^2^_partial_ = 0.27] which was due to women responding more quickly than men (703 vs. 765 ms, respectively). Gender also interacted with Age [*F*_(1,28.6)_ = 12.77, *p* < 0.01, η^2^_partial_ = 0.31], and there was also a Gender x Age x VAS-F interaction [*F*_(1,53.2)_ = 4.87, *p* < 0.05, η^2^_partial_ = 0.08]. This is shown in Figure 2 and was due to a positive relationship between age and RT in men (older individuals responded with longer latencies) and a negative relationship between age and RT for women (older individuals responded with shorter latencies). As Figure 2 shows, these relationships were exacerbated as fatigue increased. No other effects or interactions were significant in the RT data.

For the accuracy data, the only significant effect was the interaction between Gender and Age [*F*_(1,23.2)_ = 5.55, *p* < 0.05, η^2^_partial_ = 0.19] which was due to a positive relationship between accuracy and age for women (coefficient = 0.0009) and a negative relationship between accuracy and age for men (coefficient = –0.0048). That is, women tended to respond with greater accuracy as they aged while men tended to respond with less accuracy as they aged. No other main effects or interactions were significant.

### Neuroimaging Results

In the neuroimaging results, we focus on effects involving VAS-F, since this study was undertaken to better understand fatigue. Results involving Gender and Age are also reported for the sake of completeness.

As Table 2 shows, there was a main effect of VAS-F in the inferior frontal gyrus and the fusiform gyrus. This was due to a negative relationship between VAS-F and brain activation in the Inferior Frontal Gyrus [η^2^_partial_ = 0.10, coefficient (i.e., slope of regression) = –0.01], and a positive relationship between VAS-F and brain activation in the Fusiform Gyrus (η^2^_partial_ = 0.06, coefficient = 0.005).

**TABLE 2:** The brain areas associated with the main effects of VAS-F and Gender, and with the interactions of Gender × VAS-F, Gender × Age, and Gender × Age × VAS-F.

Condition/Location	X	Y	Z	Vox	*F* statistic
**VAS-F**					
Inferior frontal gyrus	–43.4	40.2	–22.1	130	22.15
Fusiform gyrus	42.7	–22.0	–22.1	47	24.67
**Gender**					
Middle temporal gyrus	–57.7	–10.1	–10.2	29	18.89
Postcentral gyrus	–55.4	–10.1	17.8	29	23.38
**Gender × VAS-F**					
Superior orbital gyrus	21.2	56.9	–10.2	32	17.35
Middle orbitofrontal gyrus	–29.1	47.3	–10.2	92	34.03
Middle frontal gyrus	–53.0	13.9	45.7	38	19.89
Inferior frontal gyrus	30.7	35.4	–14.2	35	16.95
Cerebellum (Crus 1)	–29.1	–89.0	–26.1	28	19.89
**Age × VAS-F**					
Middle frontal gyrus	–17.1	47.3	25.7	29	21.87
**Gender × Age**					
Parahippocampal gyrus	18.8	–0.5	–34.1	49	26.24
**Gender × Age × VAS-F**					
Parahippocampal gyrus	16.4	–0.5	–34.1	57	26.50

*X Y Z = the location of the voxel with peak intensity in each cluster; Vox refers to the number of voxels in the cluster; F-statistic refers to the maximal F-statistic in each cluster.*

**FIGURE 3:**
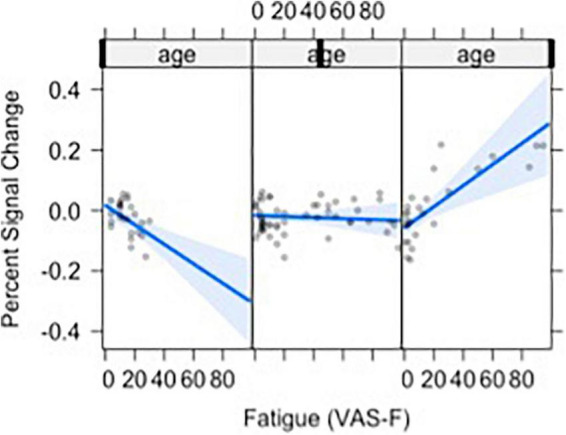
The relationship between visual analog scale of fatigue (VAS-F) and Age in the middle frontal gyrus is shown. For this plot, the age range was divided into three sections: 20–32, 33–48, 49+. This division is for exposition only and does not reflect subgrouping in the analyses of the data (age was a continuous variable in all analyses). The blue line represents the best fitting linear relationship between VAS-F and age. The 95% confidence interval around this line is plotted in lighter blue. The data are represented by gray circles.

Moreover, VAS-F interacted with Age in the middle frontal gyrus (η^2^_partial_ = 0.12, see Figure 3). This was due to younger individuals showing a negative relationship between VAS-F and brain activation, individuals aged 33–48 showing essentially no relationship between VAS-F and brain activation, and older individuals showing a positive relationship between VAS-F and brain activation. That is, in this cross-sectional sample, the relationship between self-reported fatigue and brain activation changed from negative to positive as age increased.

**FIGURE 4:**
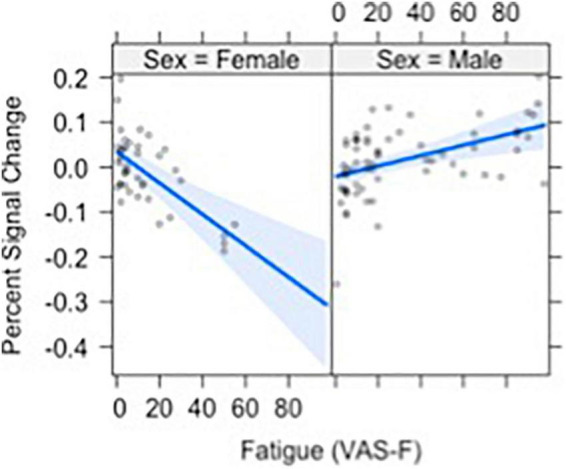
The relationship between visual analog scale of fatigue (VAS-F) and Gender in the orbito-frontal gyrus is shown. For women **(left)** the relationship between VAS-F and activation was negative; for men **(right)** it was positive. The blue line represents the best fitting linear relationship between VAS-F and age. The 95% confidence interval around this line is plotted in lighter blue.

Brain activation was also modulated by the interaction of VAS-F and Gender in orbital frontal areas, middle frontal areas and inferior frontal areas, as well as in the cerebellum (see Table 2). Figure 4 shows this interaction in the middle orbitofrontal gyrus (η^2^_partial_ = 0.28). For women, the relationship between brain activation and VAS-F was negative (coefficient = –0.004); for men, the relationship was positive (coefficient = 0.001). This pattern was comparable in the other regions showing this interaction.

Finally, there was an interaction between Gender, Age and VAS-F in the parahippocampal gyrus (η^2^_partial_ = 0.21, see Table 2). This interaction similar to the interaction between VAS-F and age in the middle frontal gyrus (see above), except that in the case of the parahippocampal gyrus, the effect of age was only evident in women: younger women showed a positive relationship between VAS-F and brain activation (coefficient = 0.020) whereas older women showed a negative relationship (coefficient = –0.077). For men, the coefficient was essentially unchanged by age (young: 0.001; old: 0.003).

## Discussion

The current study examined cognitive fatigue across age using both state and trait measures. In addition to collecting self-report measures, we conducted fMRI scans to see how activation in different brain regions related to fatigue ratings across ages 20–63. We did not find trait fatigue ratings (MFIS) to be correlated with age, however we found a negative correlation between state fatigue ratings (VAS-F) and age. We also found differences in brain activation between young adults and older adults, as well as between male and female participants at different levels of fatigue.

Our aim in using both state and trait measures in this study was to investigate whether these different dependent measures would help to explain some of the inconsistencies in the literature examining fatigue across the lifespan. While older adults reported themselves to have the same fatigue “burden” (trait fatigue) as younger adults, they nevertheless reported experiencing less fatigue while they were performing a task (state fatigue). That is, even within the same dataset, the relationship between age and fatigue was dependent upon whether state or trait fatigue measures were used, strongly suggesting that these instruments do not measure the same thing. A prior study looking at the relationship between state and trait fatigue in university students, faculty, and staff found a strong link between the two, such that greater trait fatigue predicted increased state fatigue (Manierre et al., 2020). However, this study also reported that this relationship was complicated when factors such as gender and sleep quality were introduced. The relationship between these two types of fatigue may be strongly susceptible to demographic influences, age being one such factor. It is further possible that a person’s perception of their own fatigue is clearer in the moment than when they are prompted to recall their fatigue levels over the course of a month, which could also account for some of the conflicting results in the literature.

There are several possible reasons why older individuals reported less fatigue in this study than their younger counterparts. One possibility is that older individuals had different sociodemographic characteristics than the younger participants. For example, a positive correlation has been shown between the number of children one has and one’s self-reported “tiredness” (Dolan and Kudrna, 2013). Tiredness and fatigue are different constructs, but it may nevertheless be the case that older individuals have fewer sociodemographic demands and are therefore less prone to report fatigue during task performance. It could also be the case that because older individuals will have had more experience with letter stimuli than younger individuals, the tasks used to induce fatigue were less effective in the older individuals. This hypothesis could be tested by using tasks and stimuli that were equally novel to all participants.

To our knowledge, this was the first study to explore the association between state fatigue and brain activity across age. Our results show that the relationship between VAS-F and brain activation changed across age in the middle frontal gyrus. For younger adults, brain activation in the middle frontal gyrus was negatively correlated with fatigue ratings, and as age increased this relationship changed such that for older adults the correlation was positive (greater activation was associated with higher VAS-F ratings). In a prior study comparing cognitive fatigue in multiple sclerosis and healthy control participants, a negative association was found between state fatigue ratings and activation in this region within the healthy control group, but not the MS group (Genova et al., 2013). Because healthy individuals also reported less cognitive fatigue than individuals in the MS group, this finding was consistent with the idea that the middle frontal gyrus has a moderating effect on cognitive fatigue. However, while the same effect is shown here in the younger subjects, the opposite is evident in the older individuals despite the fact that the older individuals reported less fatigue. This may suggest that the role of the middle frontal gyrus, and/or its connectivity (Wylie et al., 2020), changes over the lifespan. Future work will be required to understand this relationship; however, the results shown here strongly suggest that fatigue related brain activation changes over the lifespan and that age needs to be accounted for when investigating fatigue.

It is important to note that our sample for this paper was healthy individuals within the age range of 20–63. Cognitive fatigue is a widely reported symptom in many medical conditions and diseases that are more likely to affect older adults than younger adults (e.g., cancer, Parkinson’s Disease, stroke). For the purpose of the current study and ruling out certain potential confounding variables, we focused on healthy individuals free of neurological disease. Had we included all individuals, regardless of medical history, it is possible that we may have seen a different effect of age on fatigue due to the increased prevalence of brain injury and disease in older individuals.

We did not find a difference in trait fatigue ratings between males and females. This was surprising, given the general consensus in the literature suggesting a greater prevalence of fatigue in women than in men (Bensing et al., 1999; Avlund, 2010). One factor that may have contributed to the difference in our results relative to Bensing’s is recent societal changes. For instance, taking care of young children was a factor that influenced fatigue in women but not men in a study conducted by Bensing et al. (1999). Meanwhile, birth rates in the US have been declining since 2008 (United States Census Bureau, 2021). Furthermore, in the intervening years, there may have been a shift in gender roles toward a more equitable allocation of child care responsibilities, as evidenced by the growing number of “stay-at-home” fathers (Pew Research Center, 2014). It is uncertain whether these slight shifts are significant enough to close the apparent trait fatigue gap between genders, but factors such as childcare responsibilities should be taken into account for future studies examining fatigue between genders.

The neuroimaging data revealed differences in state fatigue-related brain activation between genders. For women, we found greater activation in the orbitofrontal gyrus to be associated with lower fatigue ratings, while for men we found greater activation to be associated with more fatigue. This raises the possibility that the orbito-frontal region, as well as the other areas of the fatigue network (Wylie et al., 2020), may respond differently to fatigue in men and women, or may have differential connectivity in men and women. For example, it is possible that this region could play a role in combatting fatigue in women, but not in men. Past studies looking at gender differences and fatigue have concentrated primarily on self-report measures (Butt et al., 2010; Engberg et al., 2017). Incorporating neuroimaging into such studies could help explain some of the differences observed. Furthermore, the differences shown here suggest that fatigue in men and women may differ, or may rely on different brain mechanisms, which suggests that treatments designed to combat fatigue after brain injury or disease may be more effective if gender is taken into account.

There were a few limitations to the current study. A cross-sectional design was used, so it was not possible to determine a change in fatigue ratings within subjects over time. Out of the 43 total subjects, ten were missing MFIS data and 11 were missing fMRI data. Furthermore, the fatigue induction paradigm used resulted in only a modest increase in fatigue over the four blocks of the task. A task that induces more fatigue should be considered for future work. Our sample size was also relatively small (*n* = 43), with a somewhat limited age range (20–63 years), so we cannot determine whether the trends found in these data continue as age increases past 63 years. Future research should explore the relationship between cognitive fatigue and brain activation in a sample of individuals with a wider range of ages. Finally, we did not consider potentially confounding variables such as quality of sleep, daytime physical activity, or caffeine intake, which may impact fatigue levels.

In conclusion, this study is the first to report the effects of gender and age on both state and trait fatigue, and also the first to report fatigue-related differences in brain activation across the lifespan and across gender during a cognitively fatiguing task. These results help to explain some of the differences reported in the literature by showing that state and trait measures of fatigue appear to measure different aspects of fatigue, and also that age and gender both appear to affect the relationship between state fatigue and brain activation.

## Data Availability Statement

The data analyzed in this study is subject to the following licenses/restrictions: The data belong to Kessler Foundation and will be made available upon request. Requests to access these datasets should be directed to GW, gwylie@kesslerfoundation.org.

## Ethics Statement

The studies involving human participants were reviewed and approved by Kessler Foundation Institutional Review Board (IRB); the IRBs at Rutgers University and the VA also reviewed and approved the studies as appropriate. The patients/participants provided their written informed consent to participate in this study.

## Author Contributions

GW and AP wrote the manuscript. HG and JD reviewed and edited the manuscript. All authors contributed to the article and approved the submitted version.

## Conflict of Interest

The authors declare that the research was conducted in the absence of any commercial or financial relationships that could be construed as a potential conflict of interest.

## Publisher’s Note

All claims expressed in this article are solely those of the authors and do not necessarily represent those of their affiliated organizations, or those of the publisher, the editors and the reviewers. Any product that may be evaluated in this article, or claim that may be made by its manufacturer, is not guaranteed or endorsed by the publisher.

## Footnotes

1
https://surfer.nmr.mgh.harvard.edu/optseq/
