# Supplementary material for: Fatigue Across the Lifespan in Men and Women: State vs. Trait
Source: Front Hum Neurosci. 2022 May 9;16:790006. doi: 10.3389/fnhum.2022.790006 (PMC9124897; doi:10.3389/fnhum.2022.790006)
Supplement: Supplementary file 1 [file Data_Sheet_1.DOCX]

Figure S1. The distribution of ages in the study sample.
